# Dual-media laser system: Nitrogen vacancy diamond and red semiconductor laser

**DOI:** 10.1126/sciadv.adj3933

**Published:** 2024-09-27

**Authors:** Lukas Lindner, Felix A. Hahl, Tingpeng Luo, Guillermo Nava Antonio, Xavier Vidal, Marcel Rattunde, Takeshi Ohshima, Joachim Sacher, Qiang Sun, Marco Capelli, Brant C. Gibson, Andrew D. Greentree, Rüdiger Quay, Jan Jeske

**Affiliations:** ^1^ Fraunhofer Institute for Applied Solid State Physics IAF, Tullastraße 72, 79108 Freiburg im Breisgau, Germany.; ^2^ Cavendish Laboratory, University of Cambridge, 19 JJ Thomson Avenue, Cambridge CB3 0HE, UK.; ^3^ TECNALIA, Basque Research and Technology Alliance (BRTA), Astondo Bidea 700, 48160 Derio, Biscay, Spain.; ^4^ Department of Materials Science, Tohoku University, Aramaki aza Aoba 6-6-02, Aoba-ku, Sendai 980-8579, Japan.; ^5^ National Institutes for Quantum Science and Technology (QST), 1233 Watanuki, Takasaki, Gunma 370-1292, Japan.; ^6^ Sacher Lasertechnik GmbH, Hedwig-Jahnow-Str. 12, D-35037 Marburg, Germany.; ^7^ ARC Centre of Excellence for Nanoscale BioPhotonics, School of Science, RMIT University, Melbourne, VIC 3001, Australia.

## Abstract

Diamond is a potential host material for laser applications due to its exceptional thermal properties, ultrawide bandgap, and color centers, which promise gain across the visible spectrum. More recently, coherent laser methods offer improved sensitivity for magnetometry. However, diamond fabrication is difficult in comparison to other crystalline matrices, and many optical loss channels are not yet understood. Here, we demonstrate a continuous-wave laser threshold as a function of the pump intensity on nitrogen-vacancy (NV) color centers. To achieve this, we constructed a laser cavity with both an NV diamond medium and an intracavity antireflection-coated diode laser. This dual-medium approach compensates intrinsic losses of the cavity by providing a fixed additional gain below threshold of the diode laser. We observe a continuous-wave laser threshold of the laser system and linewidth narrowing with increasing green pump power on the NV centers. Our results are a major development toward coherent approaches to magnetometry.

## Teaser

Combining laser media allows for quantum emitter diamond laser systems.

## INTRODUCTION

Diamond is an interesting material for lasers because of its wide bandgap, high optical transparency and thermal conductivity, both as an active medium for Raman lasing (*1*) and a well-suited host for color centers as a laser medium (*2*–*4*). One of the most famous and well-characterized color centers is the negatively charged nitrogen-vacancy (NV) center in diamond due to its atom-like energy-level structure and long coherence time at room temperature. NV centers are used in a wide range of applications from quantum information (*5*) to sensing applications for temperature (*6*), orientation and angular velocity (*7*), magnetic signature of biological samples (*8*), and electric (*9*) or magnetic fields (*10*–*14*).

Most of these sensor concepts measure the response of the fluorescence signal to environmental parameters. The magnetic sensing approach is based on the Zeeman effect of the energy levels of the NV centers. The precision of fluorescence readout depends on the amount of collected light and its response to the measured quantity, i.e., the measurement contrast. There are different approaches to increase the collection efficiency such as microlenses (*15*) or microcavities and waveguides for better coupling (*16*, *17*). A substantial improvement of the sensitivity of magnetic field measurements can be achieved by laser threshold magnetometry (LTM) (*18*). In this measurement concept, stimulated emission from the NV center itself is used as an active gain medium of a laser cavity. This allows to benefit from a magnetically dependent laser threshold, achieving high measurement contrast (ideally close to 100%). In addition, the output signal is amplified through the optical nonlinearity of the laser cavity. Because the cavity output is coherent (rather than incoherent as in conventional fluorescence-based measurements), it is also easier to monitor in the far field.

The idea of using cavities to enhance sensing has previously been realized in cavity-enhanced absorption spectroscopy (*19*, *20*), where materials are purely passive and not optically pumped. The cavity increases the effective optical path length in the material. An optically pumped in tracavity element is used in optical amplifiers (*21*, *22*). LTM combines these two concepts to create cavity-enhanced sensing via variable optical gain.

Theoretically, LTM has been projected to reach a dc sensitivity as low as $\sim \text{1fT}/\sqrt{\mathrm{Hz}}$ (*18*). For a comparable fluorescence-based magnetometer, a shot noise sensitivity in the range of a $\text{pT/}\sqrt{\mathrm{Hz}}$ can be estimated (see the Supplementary Materials). Experimentally, the best demonstrated sensitivity with a fluorescence-based approach has now reached the regime of sub-$\text{pT/}\sqrt{\mathrm{Hz}}$ (*23*–*25*). This indicates the great potential of the concept of LTM not only for NV magnetometry but also for other applications.

Different realizations of LTM systems have been proposed. Dumeige *et al.* (*26*) proposed an implementation using an NV-diamond as an optical absorber within an external laser cavity with an additional active laser medium. They project a theoretical sensitivity of $\text{700 fT}\sqrt{\text{Hz}}$ (*26*). Another theoretical approach uses the absorption of the pump laser within an external Fabry-Perot cavity. Hereby, the sensitivity can be estimated to 300 to 20 $\text{fT}/\sqrt{\text{Hz}}$ (*27*, *28*). Nair *et al.* (*29*) report on a theoretical investigation of an NV-diamond Raman laser and calculate a sensitivity of 1.62 $\text{pT}/\sqrt{\text{Hz}}$.

Experimentally, the stimulated emission from NV centers was demonstrated in 2017 (*30*), followed by a demonstration of NV-light amplification in a fiber cavity (*31*). Material characterization and optimization (*32*–*34*) were necessary to enable further improvements in material and cavity design. Hahl *et al.* (*35*) demonstrated light amplification in an externally seeded cavity using a red seeding laser. Magnetic field dependence with a record contrast and a projected sensitivity of $15\;\text{pT}/\sqrt{\text{Hz}}$ was measured, demonstrating a one-order-of-magnitude improvement in sensitivity over spontaneous emission fluorescence sensing.

In 2021, Savvin *et al.* (*36*–*38*) demonstrated a pulsed NV laser system, wherein the NV centers provide the laser system gain. A continuous-wave (cw) laser can increase the signal intensity, sensitivity, and stability of a measurement considerably. For equal laser gain, the signal strength of a cw laser is higher than that of a pulsed laser due to the charge state dynamics of the NV center (*39*, *40*). In addition, a cw signal or modulated excitation is often required for sensing measurements concepts (*23*, *41*), as precise timings need to be chosen, which is in conflict with laser pulsing. A cw laser is thus essential for sensing with improved sensitivity.

Until now, in all realizations, it was not possible to show a laser system wherein the NV centers are pumped with a cw optical laser, and therefore, the stimulated emission of the NV centers themselves may be used as a sensor. This is due to several difficulties concerning NV-doped diamond as a laser-active medium: First, while diamond is a sufficiently transparent medium for optical wavelengths, NV-doped samples often show notable absorption around 700 nm (*32*) at the maximum of the fluorescence (*42*). Second, the NV center has two possible charge states, of which only the NV^−^ is suitable for sensing applications. The proportion of NV^−^ and NV^0^ depends on the excitation power. Especially at high powers, photoionization drives a transition from NV^−^ to NV^0^. Third, high powers (at pump or read-out wavelength) within the cavity lead to an “induced absorption” effect (*31*, *35*, *36*). The pump power that is necessary to overcome the laser threshold due to absorption by the NV-diamond is limited by photoionization and induced absorption effects.

Here, we propose a solution: A dual-media laser system containing both an NV-diamond sample and a red semiconductor laser within one cavity. The concept relies on the semiconductor gain element to compensate the intrinsic losses of the cavity and the diamond, so that the NV centers only have to add a small gain (or reduced net losses) to overcome the laser threshold. We present an experimental realization using a linear cavity containing a laser diode and an NV-doped diamond sample. With this, we demonstrate a cw laser threshold of an NV laser system, which we believe is an essential step toward an LTM sensor system.

## RESULTS

### Energy-level scheme

**Fig. 1.**
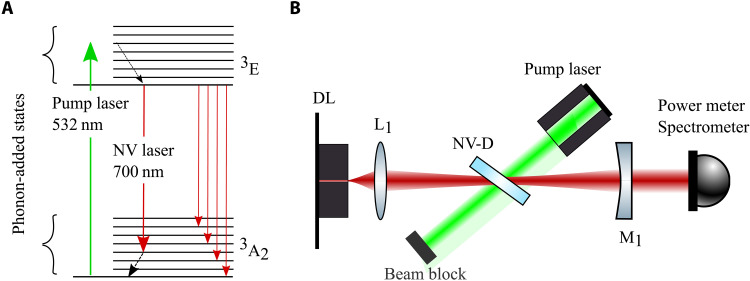
Experimental design. (**A**) Energy levels of the NV center: The green pump laser addresses a transition from the ground state to a phonon-added excited state, and the laser transition is from the excited state to a phonon-added ground state. (**B**) Sketch of the linear cavity setup: The backside mirror of the diode laser (DL) and the external outcoupling mirror (M_1_) form the cavity, the diode laser and the NV-diamond (NV-D) are the active media, and the lens (L_1_) is necessary to focus the output of the antireflection-coated side of the diode laser.

An NV laser system was proposed by Rand (*3*) in 1994. The energy levels relevant to this work are shown in Fig. 1A. The lowest-energy level is that of the electron ground state (^3^A_2_). The first excited level is within the phonon-added states of the excited electron state ^3^E. The transition can be excited with a 532 nm laser (pump laser). The energy states in the phonon-added excited states relax almost instantaneously to the lowest excited state without phonons, which is the upper laser level. The lower laser level is a phonon-added electronic ground state, which then in turn relaxes rapidly to the lowest phonon-added state. The two electronic states and their phonon-added states form a four-level laser system, which allows for lasing without the need to pump the majority of the population to the excited state to achieve population inversion, because the phonon-added ground state is depopulated quickly. This four-level laser also avoids strong photoionization toward NV^0^, which would occur for strong light fields around the zero-phonon line of 637 nm.

### Dual-media laser system

To realize a cw NV-dependent laser system, we combine an external cavity diode laser with an NV-doped diamond sample. The linear cavity setup is shown in Fig. 1B. The diode laser is antireflection-coated and emits around 690 nm wavelength. This wavelength range was chosen as a compromise between highest optical gain from the NV centers with low absorption losses of the NV-diamond sample (*32*) and technical availability of diode lasers.

The lens L_1_ collects and refocuses the diode emission. At the second focal point, the NV-diamond sample is placed. The sample is a high-pressure–high-temperature sample, which was irradiated with an electron beam and annealed in situ, according to the process described by Capelli *et al.* (*43*). The final NV concentration results to about 2 parts per million. The sample has a thickness of around 500 μm. The NV-diamond is placed at Brewster’s angle to minimize reflection losses.

The cavity end-mirror M_1_ has a reflectivity of around 95% and a convex radius of curvature of −50 mm, resulting in a mode diameter of about 200 μm at the position of the NV-diamond sample. For laser beam diagnostics, we use a power meter with an accuracy of 3% and a fiber-coupled spectrometer with a spectral resolution of 0.02 nm.

In the following experiments, both active media of the laser system will be operated near the laser threshold. To this end, the intracavity NV-diamond sample is pumped with a green laser with 532 nm emission wavelength and emits in the same red wavelength range as the diode laser. The green pump laser (single pass) and the diode laser cavity mode are carefully overlapped within the NV-diamond by optical adjustment of the green pump spot size and position while maximizing the red laser output. Additional details on the experimental realization can be found in the Supplementary Materials.

### Diode laser characteristics with fixed NV gain

**Fig. 2.**
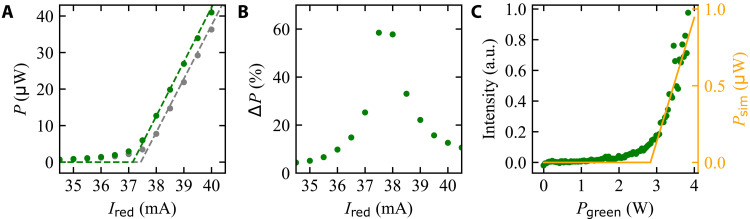
Output power of the dual-media laser system. (**A**) Output power (*P*) of the laser system as a function of the laser diode current (*I*_red_). Gray curve represents without NV gain (green pump laser turned off), and green curve denotes with fixed NV gain. Dashed lines denote the results of a simulation based on the experimental parameters. (**B**) Relative change in output power (∆*P*) between the measured values shown in (A). (**C**) Demonstration of a cw NV laser threshold: Green denotes maximum amplitude of the main laser line of the dual-media laser system as a function of green pump power on the NV-diamond sample. The laser diode is operated with a fixed-diode current below the laser threshold, which is then overcome by the NV gain. Orange denotes the result of the simulation. a.u., arbitrary unit.

As a first step, the laser threshold of the diode laser will be determined, while the diamond is inserted into the cavity but not pumped (green pump laser turned off). The gray curve of Fig. 2A shows the output power of the diode laser as a function of the laser diode current. The output power was measured with the power meter. The laser threshold is visible at a diode current of 37.45 mA. In the next step, the measurement is repeated with the diamond pumped with 532 nm green laser light (Fig. 2A, green curve). The output of the laser system is higher when the NV centers are pumped, which we assign to additional stimulated emission from the NV centers. The shift of the laser threshold shows an increase in roundtrip gain, as the fixed cavity losses are overcome at lower diode pumping. Above threshold, the curves with and without the green pump laser show comparable slopes. This is in line with a fixed increase in gain due to constant pumping of the NV centers, which simply shifts the whole curve to the left.

The dashed lines in Fig. 2A show the result of a modeling of the output power based on semiclassical laser theory using experimental parameters for the cavity and the NV-diamond sample (see the Supplementary Materials). The gray curve of Fig. 2A shows a fit to the measured values, which was used to determine the laser diode gain and saturation parameter. These parameters were then used for calculating the dashed green curve with green laser pumping. Comparing our experimental estimate for green pump intensity (4 W at 100 μm radius spot) with the value that best fits the green curve, we find that the fitted value is a factor of 1.5 lower than our estimate, which is a very good agreement between experiment and modeling of stimulated emission output, given no other fitting parameter is used for the dashed green curve. In the modeling, we neglected spontaneous emission contributions for simplicity of the model and to reduce fitting parameters. This and the multimode operation lead to the small deviation between experimental values and the simulation results just below the threshold. Details on the used model can be found in the Supplementary Materials. The good agreement of the measured output power with NV gain to the values calculated using the rate equations supports the assumed laser model of the NV center.

Figure 2B shows the relative change (∆*P*) in output power between the measured values, which was calculated as the ratio of the difference between the red power with and without green laser pumping. The influence of pumping the NV-diamond is largest at the laser threshold, where the green pump laser leads to an increase of output power by more than 50%. This confirms the principle that a small change (in this case by pumping the NV centers) in a laser signal (in this case the red laser diode) has a large relative effect at or just above the threshold. In the concept of LTM, this principle is used to amplify the small change introduced by an external magnetic field to an NV laser.

To summarize, we have demonstrated that the threshold of the red diode laser is shifted by green pumping of the NV centers. This result demonstrates a cw NV-dependent laser system, without an external seeding laser.

### NV laser characteristics with fixed diode gain

Next, we aim to achieve an NV center laser which is only assisted by the diode, i.e., a laser threshold measured as a function of the NV centers’ pump power, rather than the electric pumping of the diode. For this purpose, we set the diode current to a fixed value at 0.75 mA below threshold. This way, it contributes gain, which compensates for cavity roundtrip losses, but the actual laser operation is then achieved by pumping the NV-diamond. To ensure that we only measure the coherent laser light (in the regime of a few μW) and reject the fluorescence light, we apply spectral filtering by measuring with a spectrometer and integrating the counts within a narrow spectral window around the laser line at 687 nm, with a bandwidth of 0.04 nm. This is in addition to a spatial mode selection, as most of the fluorescence light of the NV centers can be assumed to be rejected by the small numerical aperture of the spectrometer (and the imaging optics toward it). Here, one advantage of the laser readout over fluorescence becomes apparent: The signal is entirely delivered to the detector via the collimated laser beam, while fluorescence is emitted into all directions.

**Fig. 3.**
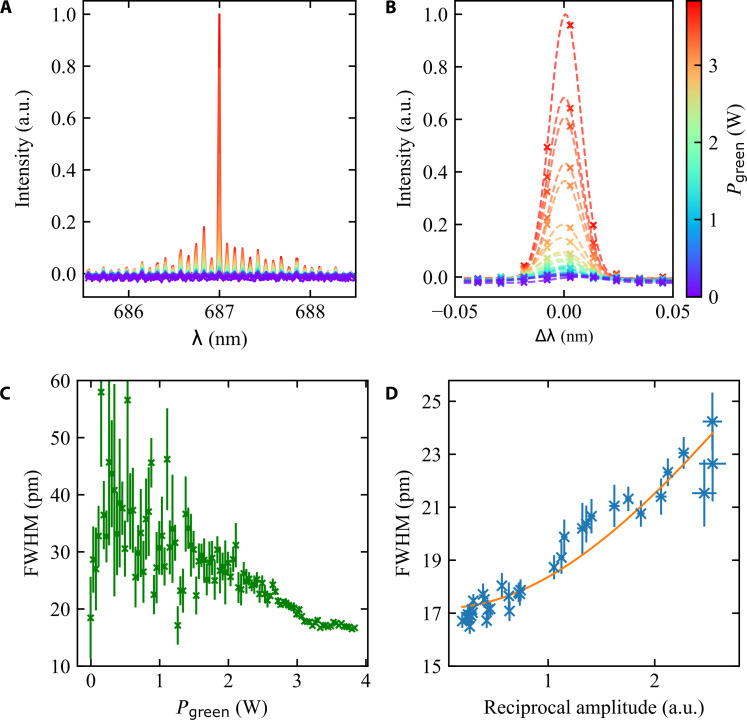
Spectral output and linewidth narrowing of the NV laser system. (**A**) Spectra of the diode-assisted NV laser system. The color corresponds to the green pump power [color scale on the right of (B)]. (**B**) Detailed view of the main laser line (measured values shown as crosses) with a fitted Gaussian profile (dashed). (**C**) Laser linewidth [full width at half maximum (FWHM), obtained from the fitted Gaussian curves] as a function of pump power. A reduction of the linewidth above the threshold (at ∼2.5 W) is visible. (**D**) Laser linewidth as a function of reciprocal amplitude (blue) of the fitted spectra shown in (B). Orange curve denotes the adapted theoretical model.

The resulting laser output as a function of the NV centers’ pump power (*P*_green_) is shown in Fig. 2C in green. The laser output spectral power shows a typical laser characteristic with a laser threshold. Below the threshold power of around 2.5 W (which corresponds to an intensity of ∼ 8 kW/cm^2^), the output is nearly constant with a low slope, while above the threshold, a linear increase is seen, with a much higher slope. The threshold appears washed out because of high intracavity losses and spontaneous emission of multimode operation (*44*), which is visible in Fig. 3A .

The laser output measurement shows of Fig. 2C significant noise above threshold, which is common in laser systems. At threshold, the laser diode gain is in balance with all intracavity losses. Because the laser diode gain is much higher than the gain of the NV centers, a small variation of the system changes the output of the laser system rapidly, which results in fluctuations.

The orange curve in Fig. 2C shows a simulation based on the same model and using the fitted parameters extracted from Fig. 2A, but with the diode current set to a fixed value of 37.36 mA (see the Supplementary Materials). Overall, the modeling shows a very consistent picture and is highly aligned with our expected parameter values. The theoretical model shows that the experimental results can well be described by stimulated emission from NV centers. We point out that the diamond can still be a net loss element due to other diamond loss channels. The observed laser threshold as a function of NV-diamond pump intensity is well explained by the given parameters of the cavity and diamond sample. The gain coefficient of the laser diode *G*_d_ is a factor ∼9 higher than the NV gain *G*_NV_. Because of additionally higher saturation values, most of the gain in the system is provided by the diode. However, we have demonstrated that cavity losses can be compensated by a second laser medium, in agreement with theoretical expectations. This allows to achieve a laser threshold behavior as a function of NV center pumping, which is theoretically predicted to be the basis for substantial sensitivity enhancement in LTM (*18*).

### Spectral characteristic

Further proof of laser action can be shown via the behavior of the emission linewidth around the threshold. Light created by stimulated emission amplifies the laser mode with a spectral characteristic: As the stimulated emission rate is proportional to the light intensity of the mode, the intense spectral center of the mode is amplified more strongly than its edge. This leads to an effective narrowing of the emission line when lasing occurs with increasing pump power (*45*, *46*). We investigate this behavior in our system.

For this, we analyze the measured spectra as a function of green pump power. Each individual spectrum is shown in Fig. 3A with a color corresponding to the pump power of the green laser on the NV-diamond sample. Multiple resonance lines can be seen, with the line at 687 nm being dominant, especially at higher pump powers. The resonant frequencies correspond to the mode profile of the laser diode (residual etalon effect). Figure 3B shows a zoom in around the main laser line at 687 nm. We fitted each spectrum with a Gaussian curve (dashed line). The full width at half maximum (FWHM) obtained from the fit is shown in Fig. 3C as a function of the green pump power on the NV-diamond sample. The fitted amplitude of the laser line is similar to the integrated spectral intensity as shown in Fig. 2C. Below the laser threshold, the spectrum shows strong fluctuations, which results in deviations of the laser linewidth. At pump powers above 2 W, the laser linewidth shows a steady decline and reaches a value of about 20 pm, which is the resolution limit of the spectrometer. The reduction of the laser linewidth above threshold is caused by the pumping of the NV centers, as the laser diode current is kept constant during the measurement. This narrowing of the linewidth shows the transition from spontaneous to stimulated emission at the laser threshold and furthermore confirms the onset of self-sustained cw lasing operation of the dual-media laser system.

The theoretical expectation of linewidth narrowing is an FWHM, which scales as the inverse of the amplitude, FWHM ∝ 1/*P*_out_ (*47*, *48*). In our case here, the spectral resolution of the spectrometer needs to be taken into account, as a convolution of the laser line with the instrument function further broadens the spectral linewidth and slightly distorts the linear relationship. As a model function for the spectral linewidth, we use $\text{FWHM}=\sqrt{{\left(p_{1}/P_{\text{out}}\right)}^{2}+{p_{2}}^{2}}$, where *p*_1*/*2_ are fit parameters and *p*_2_ represents the spectrometer resolution. To compare our experimental findings with the theoretical expectation, (Fig. 3D shows the spectral linewidth (FWHM) as a function of the reciprocal peak amplitude (extracted from Fig. 3B ) above the laser threshold as blue crosses. The fitted theoretical function is shown in Fig. 3D ) (orange curve). From the fit, we receive a spectral resolution of the spectrometer of *p*_2_ = 17 pm, which fits well to our expectation. The good agreement between measured linewidths and fitted model function is further evidence for the laser characteristic of the dual-media laser system.

## DISCUSSION

We have demonstrated a cw laser threshold as a function of NV center pump intensity using a dual-media laser cavity, which combines the two active media NV diamond and an external cavity semiconductor laser diode in the same laser cavity. We achieve this laser threshold by providing fixed additional gain from the semiconductor laser that offsets cavity losses and is operated below threshold. The laser diode defines the wavelength, where NV optical gain can create a self-sustained cw laser. In our experiments, we demonstrate a dual-media laser system, wherein the threshold of the laser system is overcome with NV pumping.

As a first step, we have shown that the laser threshold of the red diode laser decreases if the NV centers are pumped with a green laser. A high contrast of more than 50% of the diode laser system with or without pumping of the NV-diamond was demonstrated.

We then further explored the cw NV laser system by varying the NV centers’ pump power while keeping the diode laser current at a fixed value below threshold. By pumping the NV centers, the threshold of the laser system is overcome. This cw measurement of a threshold of an NV-dependent laser system demonstrates new prospects for diamond color centers as an active medium within a combined dual-media laser system. We also demonstrate the concept of realizing a quantum emitter laser from a material with insufficient gain or net losses by providing additional gain with a second laser medium, which can be adapted for other quantum emitters. Our results are a substantial step toward LTM. For this measurement concept, which promises notable improvements of sensitivity, a laser threshold is an essential advance as this is a prerequisite to realizing close to 100% contrast by shifting the laser threshold via magnetic fields (*18*). Modeling by semiclassical laser rate equations further confirms an increased NV gain and the laser characteristic of the dual-media laser system.

Spectrally resolved measurements of the NV laser system emission also show a spectral narrowing with increasing output power in accordance to laser theory and confirms the transition from spontaneous emission to stimulated emission at the laser threshold. Comparing our results to a previous demonstration of pulsed NV lasing, we note that our laser threshold at pump intensities of around 8 kW/cm^2^ is several orders of magnitude lower than in the pulsed case (5 × 10^7^ W/cm^2^) (*36*). This is highly relevant considering that in a previous publication, cw gain was shown to reduce with increasing pump intensities (*35*). This is an indication that the loss mechanism in the cw case might have a temporal onset that can be avoided by pulsed pumping. However, this also raises the question whether pulsed lasing requires a slow repetition rate or a time without pumping between pulse trains to avoid or recover from the loss mechanism. In that case, pulsed lasing would be in conflict with spin polarization as the basis for NV sensing. A commensurate study of time scales of the optical gain and loss elements could provide further insight into these questions.

Future implementations will benefit from improved NV-diamond material, which is an active field of research, promising further improvements in the aim to achieve high NV*^−^* densities and gain, low absorption, and narrow ensemble magnetic resonance linewidths (*35*, *42*). Using a different assisting laser medium—such as a surface emitting laser—will notably lower coupling and intrinsic material losses and provide long-term stability and allow to adapt and improve the laser cavity as well as reduce the pumping power required to achieve transparency in the second laser medium. An improved finesse and a higher contribution of NV-diamond gain to the total gain will lead to a higher sensitivity to the optical state of the diamond, in addition to a higher output power. These improvements would allow to use the laser threshold for LTM and realize the strong predicted sensitivity improvements as it would allow to read out the spin state of the NV center and perform magnetic field sensing via a shift in the laser threshold induced by the external magnetic fields.

For LTM at an emission wavelength of 690 nm, NV gain is required for magnetic sensing applications because possible (pump-dependent) loss channels are not sensitive to the Zeeman effect. However, using a second semiconductor gain medium allows for achieving a laser threshold without the need to achieve net positive gain in the NV diamond, i.e., again that is higher than all absorption losses and other cavity losses. This allows to explore a large new space of material and conceptual parameters in LTM. Furthermore, we would like to mention that for lower pump wavelength ranges, a magnetic field dependence of the absorption of the NV centers has been successfully demonstrated (*27*). Another approach is to use the magnetic field–dependent absorption of the NV centers at 1042 nm wavelength, involving the singlet state (*26*, *49*).

Our results provide the step from pulsed to cw lasing and from externally seeded to self-sustained NV-dependent lasing with fixed-gain assistance by the diode laser. This provides a basis for using the optical nonlinearity of the laser cavity to enhance NV sensing and promises new sensitivity records as the self-seeding allows for high contrast and signal intensity. In addition to LTM, other applications of NV-based quantum sensing, which are now typically based on fluorescence, and qubit readout may benefit from a coherent readout of stimulated emission from NV centers.

## Supplementary Materials

### This PDF file includes:

Sections S1 to S4 Figs. S1 and S2 References
