# Supplementary material for: Dual-media laser system: Nitrogen vacancy diamond and red semiconductor laser
Source: Sci Adv. 2024 Sep 27;10(39):eadj3933. doi: 10.1126/sciadv.adj3933 (PMC11430453; doi:10.1126/sciadv.adj3933)
Supplement: Supplementary file 1 — Sections S1 to S4 Figs. S1 and S2 References [file sciadv.adj3933_sm.pdf]

Science Advances
AAAS

Supplementary Materials for

Dual-media laser system: Nitrogen vacancy diamond and red semiconductor laser

Lukas Lindner *et al.*

Corresponding author: Email: Lukas Lindner, lukas.lindner@iaf.fraunhofer; Jan Jeske, jan.jeske@iaf.fraunhofer.de

Sci. Adv. **10**, eadj3933 (2024)
DOI: 10.1126/sciadv.adj3933

This PDF file includes:

Sections S1 to S4
Figs. S1 and S2
References

S1. Experimental-Setup

The diode laser (DL, Sacher Lasertechnik SAL-0690-025) is an anti-reflection coated AlGaInP diode and emits around 690 nm wavelength. The diode has an active length of around 0.7 mm.

The lens L_1 which collects and refocuses the diode emission has a focal length of 2.75 mm. The NV-diamond sample is placed at the second focal point and oriented in its Brewster's angle to minimize reflection losses. The emission from the laser diode shows a longitudinal multimode structure from which we can select one by introducing a small misalignment of the diamond sample to its Brewster's angle, creating a residual etalon effect.

The outcoupling mirror M_1 has a reflectivity of around 95% and a radius of curvature of $r = -50$ mm. The cavity is about 200 mm long, the beam waist has a diameter of about 200 µm at the position of the NV-diamond sample. A calculated beam caustic is shown in figure S1.

To measure the laser output of the dual laser system, we use a power meter (Thorlabs S130C) with an accuracy of 3% and a fiber-coupled spectrometer (Princeton Instruments, HRS-750)

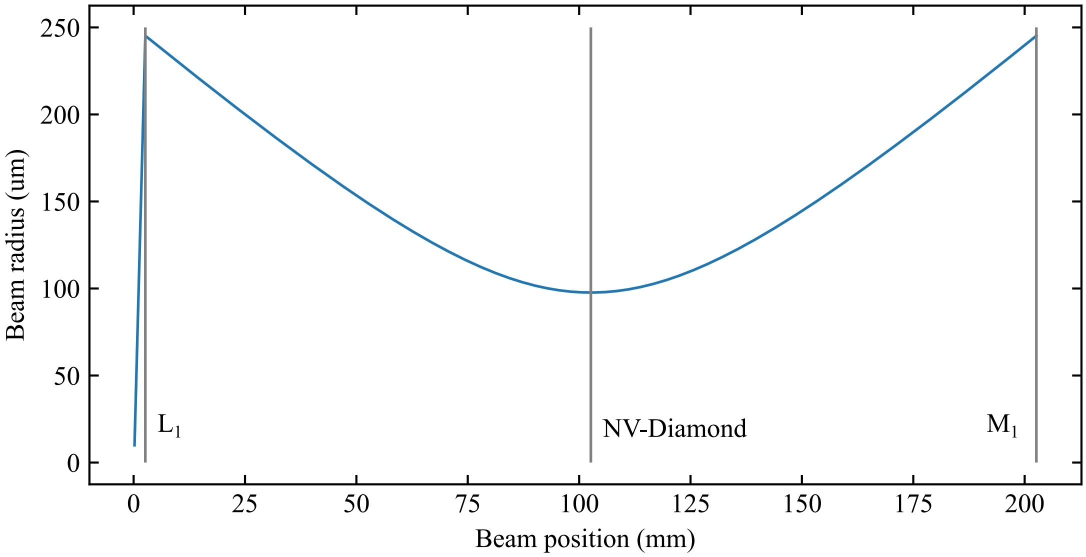

Figure S1: Calculated beam caustic of the external diode laser cavity.

with a spectral resolution of 0.02 nm. The NV-diamond sample is pumped with a green laser (Laser Quantum, axiom 532) with 532 nm emission wavelength.

S2. Diamond sample

The sample is a high-pressure-high-temperature (HPHT) Type 1b diamond sample from Element Six. The surfaces have a roughness of around 2 nm and 0.5 nm. The sample was irradiated with an electron beam (2 MeV) and a total fluence of 10^{18} e/cm^2 and annealed *in situ* with 740 °C, according to the process described by Capelli *et al.* (*43*). The diamond is 496 µm thick. The final NV-concentration results to about 2 ppm. A sample from the same batch has a concentration of P1 centers, i.e. single substitutional nitrogen, also called N_s^0 of 297.7 ppm.

A photoluminescence spectrum of the NV-diamond sample is shown in figure S2 a), which was measured under excitation with 532 nm wavelength in a self-built confocal measurement setup. This spectrum shows a clear NV^--signature and no evidence of NV^0. The absorption spectrum of the NV-diamond sample is shown in figure S2 b). This spectrum was measured using an UV-Vis absorption spectrometer. For the laser wavelength of 690 nm, the attenuation is around 4%.

S3. Theoretical sensitivity of fluorescence-based NV magnetic sensing

To calculate the theoretical DC shot noise sensitivity for a fluorescence-based NV center magnetometer, we use the following formula (*50*):

$$\eta_{DC} = \frac{4}{3\sqrt{3}} \frac{h}{g_e \mu_B} \frac{\Delta\nu}{C\sqrt{N}} \tag{S.1}$$

Here, $\Delta\nu$ is the full width half maximum linewidth of the optically detected magnetic resonance, C is the contrast and N the number of photons per second in the measurement, h is

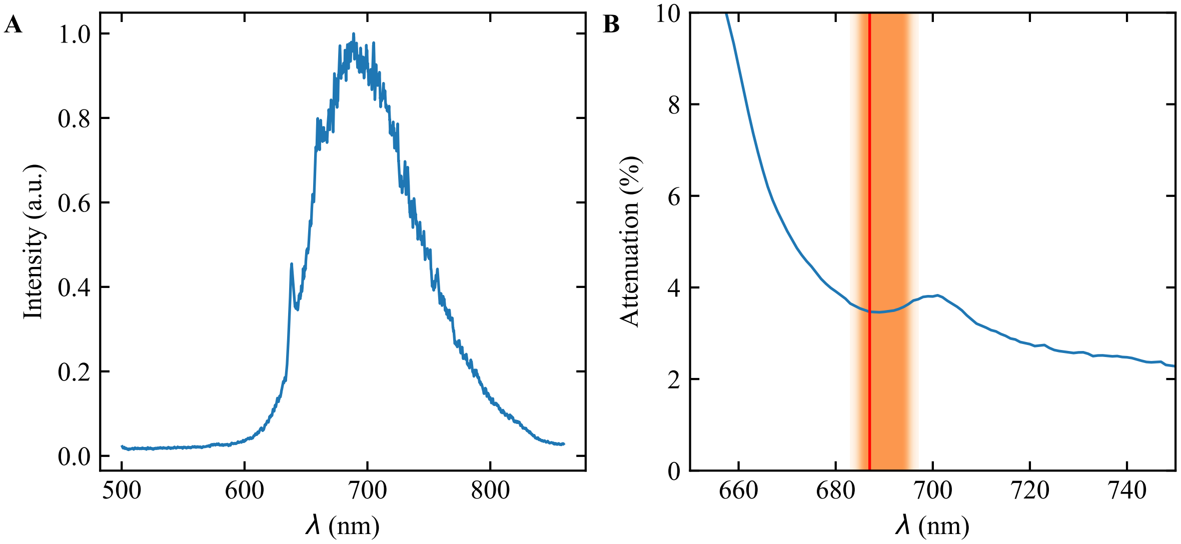

Figure S2: Optical properties of the diamond. **(A)** Photoluminescence spectrum of the NV-diamond sample, measured with a self-built confocal measurement setup. **(B)** Absorption spectrum of the NV-diamond sample, measured with a UV-Vis absorption spectrometer (PerkinElmer Lambda950).

the Planck constant, g_e is the Landé factor and μ_B the Bohr magneton. In their theoretical calculation of the sensitivity for LTM, Jeske *et al.* (*18*) use values from Acosta *et al.* (*51*) for the diamond sample: $T_2^* = 181$ ns, an NV concentration of 16 ppm and a diamond volume of 1 mm^3. These can be used to estimate the sensitivity of fluorescence-based NV magnetic sensing: We furthermore use the same green laser pump rate of $\Lambda = 10.4$ MHz, and Rabi frequency of $\Omega = 6.1$ MHz. In this regime the linewidth is determined by the power-broadening of the microwave driving and we estimate from Fig. 2b in (*18*) for a Rabi driving of $\Omega = 6.1$ MHz an FWHM linewidth of 80 MHz. With an estimated contrast of 5%, a spontaneous emission rate of $\Gamma = 1/(12\,\text{ns})$, an excited state population of $p_{exc} = \Lambda/(\Lambda + \Gamma) = 11\%$ and a collection efficiency of 1% of all light emitted by the NV centers, a theoretical sensitivity of around 2700 fT/$\sqrt{\text{Hz}}$ can be estimated.

S4. Modeling of the cavity

To model the output power of the cavity, we set up a set of coupled differential equations for the number of cavity photons, the NV centers and the diode emitters. Analogous to the paper by Jeske *et al.* and Hahl *et al.* (*18, 35*) we set up the semi-classical laser rate equations for the NV-center and add the diode gain. The number of cavity photons per NV center n is described by the differential equation:

$$\dot{n} = G_{\mathrm{d}}(p_{\uparrow} - p_{\downarrow})n + G_{\mathrm{NV}}(\rho_{22} - \rho_{33})n - \kappa n. \tag{S.2}$$

On the right hand side of the above equation, the first term describes the gain from the diode, the second term the gain from the NV centers and the third term the cavity losses. We model the NV centers as three-state emitters neglecting any differentiation between spin states where state 1 is the ground state, state 2 is the excited state and state 3 is the phonon-added ground state, which corresponds to the phonon sideband emission which is resonant with the cavity and at the wavelength of the diode emission:

$$\dot{\rho}_{11} = -\Lambda_{12}\rho_{11} + L_{21}\rho_{22} + L_{31}\rho_{33} \tag{S.3}$$

$$\dot{\rho}_{22} = \Lambda_{12}\rho_{11} - (L_{21} + L_{23})\rho_{22} - G_{\mathrm{NV}}(\rho_{22} - \rho_{33})n \tag{S.4}$$

$$\dot{\rho}_{33} = L_{23}\rho_{22} - L_{31}\rho_{33} - G_{\mathrm{NV}}(\rho_{33} - \rho_{22})n \tag{S.5}$$

The populations are described by ρ_{ii}, the green pumping of the NV-center is given by $\Lambda_{12} = I\sigma/\hbar\omega$ which connects directly to the green pump intensity I via the absorption cross section $\sigma = 0.95 \times 10^{-20}\,\mathrm{m}^2$ (*40*) and the photon energy $\hbar\omega$. The NV center's internal transition rates L_{ij} and the gain coefficient G_{NV} are defined analogous to Jeske *et al*. (*18*) based on an estimated NV number of 5.9×10^{12} in the diamond volume of the beam and a total cavity volume of $34\,\mathrm{mm}^3$ we find $G_{\mathrm{NV}} = 102\,\mathrm{MHz}$. We furthermore assume a cavity finesse of 15

yielding a cavity loss rate of κ =310 MHz, which includes round-trip losses such as absorption, scattering, or losses due to birefringence.

We model the diode emission with a two level model:

$$\dot{p}_\uparrow = \Lambda_\mathrm{I} p_\downarrow - R p_\uparrow - G_\mathrm{d} n (p_\uparrow - p_\downarrow) \tag{S.6}$$

$$\dot{p}_\downarrow = -\Lambda_\mathrm{I} p_\downarrow + R p_\uparrow + G_\mathrm{d} n (p_\uparrow - p_\downarrow) \tag{S.7}$$

Here, the electric diode pumping is described by the pump rate $\Lambda_\mathrm{I} \propto I_\mathrm{red}$ proportional to the diode current I_red. The spontaneous recombination rate of the charge carriers in the diode is described by the rate R and the diode gain by G_d. These are the fitting parameters for the model. The model does not consider any other pump-dependent loss channels such as NV^- to NV^0 ionisation or induced absorption (*31, 35, 36*).

We solve the differential equations for the steady state solution where all time-dependent variables are constant and time derivatives are zero. This allows to solve the equations for the NV center populations (and equivalently the diode emitter populations) independently as a function of the unknown but constant parameter n. These solutions can then be inserted into the differential equation for n which can then be solved analytically.

We then plot the resulting solution and adapt the unknown diode parameters Λ_I, G_d and R to best fit the experimental results. We set the diode gain coefficient to $G_\mathrm{d} = 3 \cdot \kappa$ which is higher than the cavity losses to ensure the ability to achieve lasing with the diode alone. This value is about 9 times higher than the NV gain G_NV. In the modeling we find that changing diode recombination rate and R and the diode pumping rate Λ_I by the same factor only scales the output power without changing the shape of the curves in figure 2 a). They are effective coefficients representing all influence of the total gain, such as the emitter density and gain medium thickness of the diode. To match our experimentally observed output power scale we set R =480 Hz. We then fit the correct threshold of the laser diode without any green pumping,

i.e. the gray curve in figure 2 a) by setting $\Lambda_I = (2.56 \times 10^4) \cdot I_{\mathrm{red}}$ Hz/mA. With these values we have modeled and set the laser output of the diode without regarding the NV center emission yet.

To model the NV center gain, i.e. the green curve in figure 2 a), we now test our expected value for the pumping rate with the value which best fits the experimental data. Our estimated value for a pump power of 4 W and laser spot size with radius 140 µm is $\Lambda_{12} = I\sigma/\hbar\omega$ =2.1 MHz. The value which we chose to best fit the data and produce the green modeling curve in figure 2 a) was 2.8 MHz, which is in extremely good agreement with our estimate given that there was no further fitting parameter. The resulting inversion density is limited due to the low pumping rate. While one could think that this could be improved by pumping with increased green laser power, a previous study by Hahl et al. (*35*) has shown that there is a maximum of NV gain with green pump power and thus we did not aim to increase the green laser power in the setup.

With this model we can also calculate the laser output as a function of green pumping power, for the case of fixed diode current and gain as shown in figure 2 c) and check for consistency of our model. The only fitting parameter in this case is the value of the diode current which was experimentally chosen to be just below the threshold. We find that the precise position of the laser threshold as a function of green pump power is very dependent on the precise value of the diode current. A value of 37.36 mA best represents the experimental data and produces the modeling curve in figure 2 c). This value is very consistent with figure 2 a) as it is below threshold for the curve without green pumping and above threshold for the curve with green pumping. The value is also consistent with a value close to but just below threshold without green pumping.

REFERENCES

1. R. J. Williams, O. Kitzler, Z. Bai, S. Sarang, H. Jasbeer, A. McKay, S. Antipov, A. Sabella, O. Lux, D. J. Spence, R. P. Mildren, High power diamond Raman lasers. *IEEE J. Sel. Top. Quantum. Electron.* **24**, 10.1109/JSTQE.2018.2827658 (2018).

2. S. C. Rand, L. G. Deshazer, Visible color-center laser in diamond. *Opt. Lett.* **10**, 481–483 (1985).

3. S. Rand, *Properties and growth of diamond* (INSPEC, 1994), pp. 235–239.

4. I. A. Dobrinets, V. G. Vins, A. Zaitsev, *HPHT-treated diamonds: Diamonds forever* of *Springer series in materials science* (Springer, 2013), vol. 181.

5. J. Wrachtrup, F. Jelezko, Processing quantum information in diamond. *J. Phys. Condens. Matter* **18**, S807–S824 (2006).

6. P. Neumann, I. Jakobi, F. Dolde, C. Burk, R. Reuter, G. Waldherr, J. Honert, T. Wolf, A. Brunner, J. H. Shim, High-precision nanoscale temperature sensing using single defects in diamond. *Nano Lett.* **13**, 2738–2742 (2013).

7. V. V. Soshenko, S. V. Bolshedvorskii, O. Rubinas, V. N. Sorokin, A. N. Smolyaninov, V. V. Vorobyov, A. V. Akimov, Nuclear spin gyroscope based on the nitrogen vacancy center in diamond. *Phys. Rev. Lett.* **126**, 197702 (2021).

8. L. T. Hall, G. C. G. Beart, E. A. Thomas, D. A. Simpson, L. P. McGuinness, J. H. Cole, J. H. Manton, R. E. Scholten, F. Jelezko, J. Wrachtrup, S. Petrou, L. C. L. Hollenberg, High spatial and temporal resolution wide-field imaging of neuron activity using quantum NV-diamond. *Sci. Rep.* **2**, 401 (2012).

9. F. Dolde, H. Fedder, M. W. Doherty, T. Nöbauer, F. Rempp, G. Balasubramanian, T. Wolf, F. Reinhard, L. C. L. Hollenberg, F. Jelezko, J. Wrachtrup, Electric-field sensing using single diamond spins. *Nat. Phys.* **7**, 459–463 (2011).

10. J. R. Maze, P. L. Stanwix, J. S. Hodges, S. Hong, J. M. Taylor, P. Cappellaro, L. Jiang, M. V. G. Dutt, E. Togan, A. S. Zibrov, A. Yacoby, R. L. Walsworth, M. D. Lukin, Nanoscale magnetic sensing with an individual electronic spin in diamond. *Nature* **455**, 644–647 (2008).

11. Y. Ruan, D. A. Simpson, J. Jeske, H. Ebendorff-Heidepriem, D. W. M. Lau, H. Ji, B. C. Johnson, T. Ohshima, S. Afshar V, L. Hollenberg, A. D. Greentree, T. M. Monro, B. C. Gibson, Magnetically sensitive nanodiamond-doped tellurite glass fibers. *Sci. Rep.* **8**, 1268 (2018).

12. D. A. Simpson, J.-P. Tetienne, J. M. McCoey, K. Ganesan, L. T. Hall, S. Petrou, R. E. Scholten, L. C. Hollenberg, Magneto-optical imaging of thin magnetic films using spins in diamond. *Sci. Rep.* **6**, 22797 (2016).

13. T. Wolf, P. Neumann, K. Nakamura, H. Sumiya, T. Ohshima, J. Isoya, J. Wrachtrup, Sub picotesla diamond magnetometry. *Phys. Rev. X* **5**, 041001 (2015).

14. J. M. Taylor, P. Cappellaro, L. Childress, L. Jiang, D. Budker, P. R. Hemmer, A. Yacoby, R. Walsworth, M. D. Lukin, High-sensitivity diamond magnetometer with nanoscale resolution. *Nat. Phys.* **4**, 810–816 (2008).

15. L. Robledo, L. Childress, H. Bernien, B. Hensen, P. F. Alkemade, R. Hanson, High-fidelity projective read-out of a solid-state spin quantum register. *Nature* **477**, 574–578 (2011).

16. E. R. Eisenach, J. F. Barry, M. F. O'Keeffe, J. M. Schloss, M. H. Steinecker, D. R. Englund, D. A. Braje, Cavity-enhanced microwave readout of a solid-state spin sensor. *Nat. Commun.* **12**, 1357 (2021).

17. B. J. M. Hausmann, B. Shields, Q. Quan, P. Maletinsky, M. McCutcheon, J. T. Choy, T. M. Babinec, A. Kubanek, A. Yacoby, M. D. Lukin, M. Loncar, Integrated diamond networks for quantum nanophotonics. *Nano Lett.* **12**, 1578–1582 (2012).

18. J. Jeske, J. H. Cole, A. D. Greentree, Laser threshold magnetometry. *New J. Phys.* **18**, 013015 (2016).

19. J. Morville, S. Kassi, M. Chenevier, D. Romanini, Fast, low-noise, mode-by-mode, cavity enhanced absorption spectroscopy by diode-laser self-locking. *Appl. Phys. B* **80**, 1027–1038 (2005).

20. T. Gherman, D. Romanini, Mode–locked cavity–enhanced absorption spectroscopy. *Opt. Express* **10**, 1033–1042 (2002).

21. G. Stewart, K. Atherton, H. Yu, B. Culshaw, An investigation of an optical fibre amplifier loop for intra-cavity and ring-down cavity loss measurements. *Meas. Sci. Technol.* **12**, 843–849 (2001).

22. I. Paiss, S. Festig, R. Lavi, Narrow-linewidth optical parametric oscillator with an intracavity laser gain element. *Opt. Lett.* **21**, 1652–1654 (1996).

23. C. Zhang, F. Shagieva, M. Widmann, M. Kubler, V. Vorobyov, P. Kapitanova, E. Nenasheva, R. Corkill, O. Rhrle, K. Nakamura, H. Sumiya, S. Onoda, J. Isoya, J. Wrachtrup, Diamond magnetometry and gradiometry towards subpicotesla DC field measurement. *Phys. Rev. Appl.* **15**, 064075 (2021).

24. I. Fescenko, A. Jarmola, I. Savukov, P. Kehayias, J. Smits, J. Damron, N. Ristoff, N. Mosavian, V. M. Acosta, Diamond magnetometer enhanced by ferrite flux concentrators. *Phys. Rev. Res.* **2**, 023394 (2020).

25. S. M. Graham, A. Rahman, L. Munn, R. L. Patel, A. J. Newman, C. J. Stephen, G. Colston, A. Nikitin, A. M. Edmonds, D. J. Twitchen, M. L. Markham, G. W. Morley, Fiber-coupled diamond magnetometry with an unshielded sensitivity of 30pT/√Hz. *Phys. Rev. Appl.* **19**, 044042 (2023).

26. Y. Dumeige, J.-F. Roch, F. Bretenaker, T. Debuisschert, V. Acosta, C. Becher, G. Chatzidrosos, A. Wickenbrock, L. Bougas, A. Wilzewski, D. Budker, Infrared laser threshold magnetometry with a NV doped diamond intracavity etalon. *Opt. Express* **27**, 1706–1717 (2019).

27. J. L. Webb, A. F. Poulsen, R. Staacke, J. Meijer, K. Berg-Sørensen, U. L. Andersen, A. Huck, Laser threshold magnetometry using green-light absorption by diamond nitro gen vacancies in an external cavity laser. *Phys. Rev. A* **103**, 062603 (2021).

28. S. Ahmadi, H. el-Ella, A. M. Wojciechowski, T. Gehring, J. O. Hansen, A. Huck, U. L. Andersen, Nitrogen-vacancy ensemble magnetometry based on pump absorption. *Phys. Rev. B* **97**, 024105 (2018).

29. S. R. Nair, L. J. Rogers, D. J. Spence, R. P. Mildren, F. Jelezko, A. D. Greentree, T. Volz, J. Jeske, Absorptive laser threshold magnetometry: Combining visible diamond Raman lasers and nitrogen-vacancy centres. *Mater. Quantum. Technol.* **1**, 025003 (2021).

30. J. Jeske, D. W. M. Lau, X. Vidal, L. P. McGuinness, P. Reineck, B. C. Johnson, M. W. Doherty, J. C. McCallum, S. Onoda, F. Jelezko, T. Ohshima, T. Volz, J. H. Cole, B. C. Gibson, A. D. Greentree, Stimulated emission from nitrogen-vacancy centres in diamond. *Nat. Commun.* **8**, 14000 (2017).

31. S. Raman, L. J. Rogers, X. Vidal, R. P. Roberts, H. Abe, T. Ohshima, T. Yatsui, A. D. Greentree, J. Jeske, T. Volz, Amplification by stimulated emission of nitrogen-vacancy centres in a diamond-loaded fibre cavity. *Nanophotonics* **9**, 4505–4518 (2020).

32. E. Fraczek, V. G. Savitski, M. Dale, B. G. Breeze, P. Diggle, M. Markham, A. Bennett, H. Dhillon, M. E. Newton, A. J. Kemp, Laser spectroscopy of NV- and NV0 colour centres in synthetic diamond. *Opt. Mater. Express* **7**, 2571–2585 (2017).

33. T. Luo, L. Lindner, R. Blinder, M. Capelli, J. Langer, V. Cimalla, F. A. Hahl, X. Vidal, J. Jeske, Rapid determination of single substitutional nitrogen N_s concentration in diamond from UV-Vis spectroscopy. *Appl. Phys. Lett.* **121**, 064002 (2022).

34. T. Luo, F. A. Hahl, J. Langer, V. Cimalla, L. Lindner, X. Vidal, M. Haertelt, R. Blinder, S. Onoda, T. Ohshima, J. Jeske, Absorption and birefringence study for reduced optical losses in diamond with high nitrogen-vacancy concentration. *Philos. Trans. A Math. Phys. Eng. Sci.* **382**, 20220314 (2024).

35. F. A. Hahl, L. Lindner, X. Vidal, T. Luo, T. Ohshima, S. Onoda, S. Ishii, A. M. Zaitsev, M. Capelli, B. C. Gibson, A. D. Greentree, J. Jeske, Magnetic-field dependent stimulated emission from nitrogen-vacancy centers in diamond. *Sci. Adv.* **8**, eabn7192 (2022).

36. A. Savvin, A. Dormidonov, E. Smetanina, V. Mitrokhin, E. Lipatov, D. Genin, S. Potanin, A. Yelisseyev, V. Vins, NV^- diamond laser. *Nat. Commun.* **12**, 7118 (2021).

37. E. I. Lipatov, D. E. Genin, M. A. Shulepov, E. N. Tel'minov, A. D. Savvin, A. P. Eliseev, V. G. Vins, Superluminescence in the phonon wing of the photoluminescence spectrum of NV centres in diamond optically pumped at λ = 532 nm. *Quantum Electron.* **52**, 465–312 (2022).

38. V. P. Mironov, E. N. Tel'minov, D. E. Genin, E. I. Lipatov, M. A. Shulepov, A. E. Dormidonov, A. D. Savvin, A. P. Yelisseyev, V. G. Vins, Peculiarities of nitrogen-vacancy centers' superluminescence in diamond under optical pumping at 532 nm. *Appl. Phys. B* **129**, 18 (2023).

39. L. Hacquebard, L. Childress, Charge-state dynamics during excitation and depletion of the nitrogen-vacancy center in diamond. *Phys. Rev. A* **97**, 063408 (2018).

40. R. Chapman, T. Plakhotnik, Quantitative luminescence microscopy on nitrogen-vacancy centres in diamond: Saturation effects under pulsed excitation. *Chem. Phys. Lett.* **507**, 190–194 (2011).

41. J. F. Barry, J. M. Schloss, E. Bauch, M. J. Turner, C. A. Hart, L. M. Pham, R. L. Walsworth, Sensitivity optimization for NV-diamond magnetometry. *Rev. Mod. Phys.* **92**, 015004 (2020).

42. T. Luo, L. Lindner, J. Langer, V. Cimalla, X. Vidal, F. Hahl, C. Schreyvogel, S. Onoda, S. Ishii, T. Ohshima, D. Wang, D. A. Simpson, B. C. Johnson, M. Capelli, R. Blinder, J. Jeske, Creation of nitrogen-vacancy centers in chemical vapor deposition diamond for sensing applications. *New J. Phys.* **24**, 033030 (2022).

43. M. Capelli, A. Heffernan, T. Ohshima, H. Abe, J. Jeske, A. Hope, A. Greentree, P. Reineck, B. Gibson, Increased nitrogen-vacancy centre creation yield in diamond through electron beam irradiation at high temperature. *Carbon* **143**, 714–719 (2019).

44. A. Siegman, *Lasers* (University Science Books, 1986).

45. W. T. Silfvast, *Laser fundamentals* (Cambridge Univ. Press, ed. 1, 2008).

46. F. Hide, M. A. Diaz-Garcia, B. J. Schwartz, M. R. Andersson, Q. Pei, A. J. Heeger, Semi conducting polymers: A new class of solid-state laser materials. *Science* **273**, 1833–1836 (1996).

47. W. Elsasser, E. O. Gobel, Spectral linewidth of gain- and index-guided InGaAsP semicon-ductor lasers. *Appl. Phys. Lett.* **45**, 353–355 (1984).

48. W. Elsasser, E. Gobel, Multimode effects in the spectral linewidth of semiconductor lasers. *IEEE J. Quantum Electron.* **21**, 687–692 (1985).

49. N. S. Gottesman, M. A. Slocum, G. A. Sevison, M. Wolf, M. L. Lukowski, C. Hessenius, M. Fallahi, R. G. Bedford, Infrared vertical external cavity surface emitting laser threshold magnetometer. *Appl. Phys. Lett.* **124** 091110 (2024).

50. A. Dreau, M. Lesik, L. Rondin, P. Spinicelli, O. Arcizet, J.-F. Roch, V. Jacques, Avoiding power broadening in optically detected magnetic resonance of single NV defects for enhanced dc magnetic field sensitivity. *Phys. Rev. B* **84**, 195204 (2011).

51. V. M. Acosta, E. Bauch, M. P. Ledbetter, C. Santori, K.-M. C. Fu, P. E. Barclay, R. G. Beausoleil, H. Linget, J. F. Roch, F. Treussart, S. Chemerisov, W. Gawlik, D. Budker, Diamonds with a high density of nitrogen-vacancy centers for magnetometry applications. *Phys. Rev. B* **80**, 115202 (2009).
